# Gun Violence in United States: In Search for a Solution

**DOI:** 10.3389/fpubh.2014.00017

**Published:** 2014-03-03

**Authors:** Muni Rubens, Nancy Shehadeh

**Affiliations:** ^1^Health Promotion and Disease Prevention, Florida International University, Miami, FL, USA; ^2^Health Administration Program, Florida Atlantic University, Boca Raton, FL, USA

**Keywords:** gun violence, mental health, social ecological model, individual level influence, community, society

## Introduction

The Navy Yard shooting at Washington DC, with 12 victims and gunman killed, after the deadly Sandy Hook massacre, has again reopened the debate on gun-shooting violence in the United States over the last 15 years; though in reality, a total of 62 episodes in schools and other sites occurred since 1982 (1). Who could have imagined that Columbine, CO, USA (15 died) in 1999 would fail to be an anomaly and initiate a series of shootings at such schools as Red Lake High School, MN, USA (10 died), Virginia Tech, VA, USA (33 died), Chardon High School, OH, USA (3 died), and Amish School, Lancaster, PA, USA (6 died). These incidences create an unsettling atmosphere for the affected families and the empathetic public. A closer look at mass murders and shooters reveals some trends and possible interventions. Although the events in Newtown, Connecticut raised a renewed dialog on preventing similar tragedies in the future and focused the discussion on the mentally ill, violence in individuals, the ability to access mental health services, gun control, and the association between the media and violence, the shooting at Navy Yard has proven that nothing much has changed.

## Theoretical Framework of Social Ecological Model

**Figure A1:**
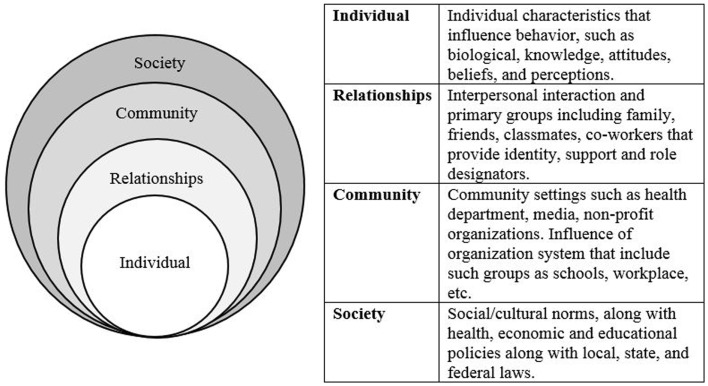
**Social ecological model levels (adapted from the framework used by the CDC to address the concept of violence)**.

From the public health perspective, the issue of gun violence could be evaluated based on the theoretical framework of social ecological model (SEM) by the Center for Disease Control and Prevention (CDC) (2). The SEM uses four levels of influence to describe a framework that identifies factors that either places a person at-risk for or guards them from being subjected to or causing a health problem such as violence. The levels of influence in the SEM structure include the individual or intrapersonal, relationships or interpersonal, community, and society (see Figure A1 in Appendix). Each level represents a key point in the process of violence, and thereby, offers an opportunity to intervene in violence for prevention. The framework also provides a tool to use in evaluation of public health issues of firearm violence.

## Components of SEM for Gun Violence Intervention

According to researchers, mental illness fails to be a sufficient reason for mass murder (3). Many individuals suffer from mental health problems, but fail to commit homicide. An individual’s attributes remain inexplicably complex. Numerous factors converge to create the rare event of public shootings (1). A more recent review found possible mental issues in individuals going on rampage shootings (4). The researchers found mental problems to be related to trauma from broken homes with prior physical or sexual abuse, psychotic behavior with symptoms of schizophrenia or schizotypal personality disorders with paranoid delusions and psychopathic behavior of narcissism, a lack of empathy, a lack of conscience, and sadistic behavior. Violence occurred more frequently with individuals with a history of being socially ostracized, exhibiting poor anger management, a fascination with violence, and possessing a strong attraction and easy access to guns. Unfortunately, the perpetrators rarely perceive an individual need for counseling or mental health services.

### Individual or intrapersonal level influence

A forensic scientist describes some of these mass murders as “pseudocommandos” who massacre in public, formulate their attack well in advance and arrive with an arsenal of firearms at the scene (5). Withdrawn demeanor, social isolation, and poor impulse control appear in individuals perpetuating gun violence (3). The individuals possess a long history of strong feelings of anger and resentment from a lifetime or long interval of collecting injustices. Some mass murderers become preoccupied with themes of violence and death that come to light in writings, drawings, threats, and bullying. The person goes on a highly personal mission to obtain revenge from a rejecting world and this individual leaves a communication of some kind to the public or news media (6). Providing mental health services to individuals with risks for harming others is an example of intervention at individual or intrapersonal level of SEM.

### Relationships or interpersonal level influence

In many of the multiple victim incidents, the perpetrator made comments about an attack to more than one other person before its occurrence, but the information known to the peers or significant others failed to be passed on to an adult or followed up on by a responsible adult. Comments tend to be general in nature such as “something bad is going to happen” rather than explicit threats such as “I’m going to kill you” (4). Third parties, particularly teachers and family members, usually possess the ability to identify the individual needing help and to locate resources through such organizations as the National Alliance on Mental Illness (7, 8). In our opinion, the role of the social networks of peers and individuals could provide relationships or interpersonal level opportunity to direct an individual exhibiting the withdrawn demeanor and hints of a violent tendency into counseling or mental health services.

### Community level influence

Knoll advocates teaching compassion, non-violence, and personal responsibility at a young age in order to go beneath the problem and implement primary prevention (8). The virtue of responsibility requires cultivating the mind during growth and development. We think that both parents and teachers carry an obligation for this training. Organizations such as schools, workplaces, recreational facilities, and other social gathering places implement zero tolerance for violence. These organization are examples of community level opportunities, which can play a pivotal role in diverting these individuals into programs for anger management, counseling or other interventions, and preventing killing sprees (9).

### Society level influence

The media represents a significant communication platform that shapes the viewing or listening audience. One’s perception arises from the images on the television and the internet, photos in blogs and in newspapers, thousands of face-to-face dialogs, emails, and on Twitter and Facebook. Some perpetrators became motivated by news coverage of the infamy of previous homicidal tragedies. The news media exploited the violent and tragic acts of the murderers after the Columbine and Virginia Tech events. However, the media provided an appropriate coverage of Newtown by keeping the public informed and not glorifying or demonizing the wrongdoer (8). A further positive action by some of the news channels involved using mental health professionals to advise the public on the psychological issues to work through. Offering guidance provides a proactive method to assist teachers, parents, and others directly and indirectly affected by the social disaster (10). When leadership at the scene provides a key spokesperson to impart factual information in a timely manner to the public, the communication reduces panic and uncertainty. In our opinion, the best approach involves providing open dialog to eliminate misconceptions and reduce anxiety about topics that involve horrendous subjects like the killing of innocent children and adults. Table 1 presents the four levels of influence of SEM with possible interventions and corresponding formative research questions which could be answered.

**Table 1:** **Social ecological model of influence^a^**.

Level of influence	Examples of possible interventions	Formative research questions
Individual	Interventions targeting early parent–child relationships. These interventions can improve trust, empathy, and cognitive functioning	How effectively can aggressive tendencies be controlled in children, which are reinforced at early ages, after which youngster develop resistance to interventions?
Relationships	Interventions targeting school activities of children. These kind of interventions will provide a venue for parents to meet teachers and other children	What is the role of pro-social parenting in developing effective relationships between parents and children?
Community	Multipronged media campaign on gun violence and identifying at-risk individuals	How effective is the role of media in curbing gun violence?
Society	Increase tax on guns in accordance with social costs. Severer legislation and penalties against individuals who violate gun safety norms and indulge in gun violence	What are the impacts of gun violence control enforcement approaches on gun access policy compliance?

*^a^Adapted from the framework used by the CDC to address the concept of violence*.

## Discussion

Levine and colleagues report the United States homicide rate for 15–24 years old to be 42.7 times higher than in other high-income countries (11). Firearms represent the mechanism of injury in 83% of the homicides and males commit 86% of the lethal shootings in the United States. Unfortunately, the debate about firearms remains an emotional and political issue with the focus on bans to rapidly firing assault weapons and more back ground checks (12). The problem continues to be more fundamental than gun ownership and the Second Amendment. A paucity of scientific information exists and prevents sound judgment by the people who need to make the decisions. We think that this major problem exists at the society level due to the lack of research on firearms related to violence. Congress stipulated that no CDC injury-prevention funds or the Department of Health and Human Services funds may be utilized to advocate or promote gun control (Who calls the shots? 2012). Society desperately needs peer-reviewed and evidence based research to address even basic questions about firearm violence registration and licensing of guns to perpetrators of gun violence (13). The American Academy of Pediatrics supports the funding of research on surveillance of firearm injuries, evaluation of healthcare screening and intervention, and identifying and disseminating violence prevention resources (14). Politicians need to focus on renewed support for research into gun violence.

Controlling gun violence is a complex and formidable task. It is well established that multi-level approach is needed to end gun violence. We provided some suggestions for policymakers and practitioners based on the SEM. The association between violence and the interaction between different factors, from individual to societal, suggest that addressing risk factors or devising prevention plans across various levels of the SEM may lead to decrease in incidences like Sandy Hook massacre and Navy Yard shooting. However, we acknowledge that implementing all these suggestions at a time is practically impossible. Since we don’t have a false proof mechanism to identify and intervene with people who might be potential culprits, the initial step should be to prevent the weapons getting into the hands of these people. This initial steps could be achieved by changing the social norms on guns and implementing some immediately workable policies (15). As the norm on the propriety of driving has changed over time, there is no reason to believe that norms about guns will not change. One such norm should be keeping the guns in safe and secure places as many perpetrators used guns which were stolen. Some of the policy changes we recommend are stricter provisions in obtaining gun license and its periodic renewal. In addition, people should undergo rigorous background checks and extensive gun safety trainings before obtaining gun. However, the challenges remain and require long term solutions.
